# Cyclin-dependent kinase 5 negatively regulates antiviral immune response by disrupting myeloid differentiation primary response protein 88 self-association

**DOI:** 10.1080/21505594.2023.2223394

**Published:** 2023-06-18

**Authors:** Jian-Ping Ren, Hao-Long Cong, Li-Jie Gao, Dong-Fang Jiang, Xin-Tong Li, Yun Wang, Jiu-Qiang Wang, Tie-Shan Tang

**Affiliations:** a State Key Laboratory of Membrane Biology, Institute of Zoology, Chinese Academy of Sciences, Beijing, China; b University of Chinese Academy of Sciences, Beijing, China; c Beijing Institute for Stem Cell and Regenerative Medicine, Beijing, China; d Chinese Academy of Inspection and Quarantine, Beijing, China; e Peninsular Cancer Center, Binzhou Medical University, Yantai, China

**Keywords:** CDK5, IFNs, INNATE IMMUNITY, MyD88

## Abstract

As a member of the pattern recognition receptors (PRRs) involving in the innate immune system, Toll-like receptors (TLRs) can sense a wide range of microbial pathogens and combat infections by producing antimicrobial products, inflammatory cytokines, and chemokines. All TLRs, with the exception of TLR3, activate a signalling cascade via the myeloid differentiation primary response gene 88 (MyD88). Therefore, the activation of MyD88-dependent signalling pathway must be finely controlled. Herein, we identified that cyclin-dependent kinase 5 (CDK5) negatively regulated TLR-MyD88 signalling pathway by targeting MyD88. Overexpression of CDK5 reduced the production of interferons (IFNs), while a deficiency in CDK5 increased the expression of IFNs in response to *vesicular stomatitis virus* (VSV) infection. Mechanistically, CDK5 suppressed the formation of MyD88 homodimers, resulting in the attenuated production of IFNs induced by VSV infection. Surprisingly, its kinase activity does not play a role in this process. Therefore, CDK5 can act as an internal regulator to prevent excessive production of IFNs by restricting TLR-MyD88-induced activation of antiviral innate immunity in A549 cells.

## Introduction

As the first line of defence against pathogenic infections, innate immunity can rapidly detect, respond to, and combat pathogen invasion. The immune system has developed a wide range of pattern recognition receptors (PRRs) to detect various components of pathogens, among which the most common are Toll-like receptors (TLRs) and retinoic acid-inducible gene-I (RIG-I)-like receptors (RLRs) [1,2]. Upon recognizing the presence of invading pathogens, these PRRs are activated and induce downstream signalling proteins, resulting in the expression of effector genes, such as type I interferons (IFNs).

RLRs, such as RIG-I and melanoma differentiation-associated protein 5 (MDA5), are considered cytoplasmic receptors because of their capacity to detect viral RNA within the cytoplasm. Both RIG-I and MDA5 harbour a DExD/H-box RNA helicase domain at C-terminus and two caspase recruitment domains (CARDs) at their N-terminus. Upon sensing virus RNA with the RNA helicase domain, RIG-I and MDA5 interact with mitochondrial antiviral signalling protein (MAVS, also known as IPS−1, VISA, or CARDIF) with their CARDs domain [3–5]. Subsequently, MAVS interacts with TANK binding kinase 1 (TBK1) to stimulate type I IFN production via phosphorylating IRF3 and IκB kinase (IKK) complex to elicit inflammatory response by phosphorylating NF-κB [6]. Generally, the expression of RLRs in resting cells is limited to a low level. However, in response to interferon exposure or viral infection, the expression of RLRs increase significantly [7–9].

In contrast to RLRs, TLRs can recognize a variety of pathogens, with TLR3, TLR7, TLR8, and TLR9 primarily detecting viral nucleic acids. Except for TLR3, all TLRs transduce downstream signals through MyD88 [10]. MyD88 contains two critical domains: the Toll/Interleukin−1 receptor (TIR) domain, which mediates the interaction with TLRs, and the death domain (DD), which bridges the downstream kinases [11]. Upon recruitment to TLRs, MyD88 forms aggregates to dimerize and become activated [12,13]. Subsequently, activated MyD88 can directly or indirectly interact with interferon regulatory factor 7 (IRF7) to initiate the transcription of IFNs by promoting the translocation of IRF7 to the nucleus [14,15]. As a central adaptor in TLRs-triggered innate immune responses, the activity of MyD88 must be finely controlled. MyD88 can be ubiquitinated by several E3 ubiquitin ligases and undergo degradation [16–18]. Additionally, MyD88-mediated downstream signalling pathways can be modulated by its phosphorylation [19–21]. Besides, the activity of MyD88 can be downregulated by reducing its self-association [13].

CDK5, as a member of the CDK family, requires interaction with P35 or P39, or their truncated forms, to become activated [22], which is distinct from other CDKs [23]. Previously, CDK5 was thought to mainly function in postmitotic neurons overexpressing its co-activators P35 and/or P39 [24]. However, CDK5 and its activators have been shown to exhibit aberrant expression in various solid and haematological malignancies [25–27]. In addition to participating in brain development, insulin secretion, cell migration, myogenesis, vesicular transport, apoptosis, and senescence [28,29], CDK5 also regulates innate immunity. CDK5 has been shown to suppress regulatory T cells’ (Treg) development through specific phosphorylation of Stat3 at Serine 727 [30]. Besides regulating the differentiation and survival of T cells [31], CDK5 is also required for T cells’ activation [32]. However, the role of CDK5 in antiviral immunity, an important part of innate immunity, remains unclear.

In the present study, we identified a novel kinase-independent function of CDK5 in regulating the antiviral innate immune system. It was found that CDK5 overexpression decreased the self-association of MyD88, subsequently attenuating the expression of IFN-β which was triggered by the TLRs-MyD88 signalling pathway. Moreover, the attenuated self-association of MyD88 was not dependent on the kinase activity of CDK5. These findings suggest that CDK5 may act as an intrinsic suppressor to prevent excess production of IFNs by targeting MyD88.

## Materials and methods

### Cells and viruses

A549 and HEK293T cells were cultured in DMEM supplemented with 10% foetal bovine serum (FBS) at 37 °C and 5% CO_2_. Polyethylenimine (PEI, 1 mg/ml) or lipofectamine 2000 (Thermo Fisher Scientific Inc.) was used for plasmid transfection according to the manufacturer’s instructions. VSV-GFP was propagated in Vero cells as previously described [33]. All viruses were infected at a multiplicity of infection of 0.1 (MOI = 0.1). For Western blot experiments, the cells were infected with VSV for 2, 4, or 6 hours. 4- or 8-hour infection of VSV was enforced for qRT-PCR analysis. Additionally, for the chemical cross-linking assay, the cells were treated with VSV for 3 hours.

### Molecular cloning of related genes

cDNA encoding CDK5, P35 and MyD88 were amplified by PCR and inserted into SBP−2×Flag, pEGFP-N1, MC-HA-pCS2 or pCMV-Tag2B-Flag vectors. CDK5, P35, MyD88 were obtained from human cDNA with RT-PCR and then ligated into SBP−2×Flag, pEGFP-N1, MC-HA-pCS2 or pCMV-Tag2B-Flag vectors.

### Reagents and antibodies

The reagents used were as follows: 3-Methyladenine (3 MA; 189490, Sigma), MG132 (474790, Millipore), Roscovitine (SC−24002, Santa Cruz), zymosan (Z4250, Sigma), lipopolysaccharide (LPS; L2880, Sigma), imiquimod (IMQ; HY-B0180A, MCE). Antibodies: RIG-I (3743, CST), MDA5 (5321, CST), CDK5 (ab40773, Abcam), CDK5 (AHZ0492, Invitrogen), P65 (39369, Active Motif), phospho-p65 (Ser529; 39691, Active Motif), phospho-TBK1 (Ser172; 5483, CST), TBK1 (3504, CST), GFP-tag antibody (M20004, Abmart), phospho-IRF3 (Ser396; 4947, CST), IRF3 (11904, CST), Flag-tag antibody (F1804, Sigma), MyD88 (4283, CST), β-actin (60008–1, Proteintech), phospho-Stat1 (Tyr701; 9167, CST), Stat1 (14994, CST), Flag-tag antibody (F7425, Sigma), β-tubulin (M20005, Abmart), phospho-Stat2 (Tyr690; 88410, CST), Stat2 (72604, CST), phospho-IRAK4 (Thr345/Ser346; 11927, CST), IRAK4 (4363, CST), GAPDH (60004–1-Ig, Proteintech), GFP (A) beads (KTSM1301, KT Health) and anti-Flag M2 beads (A2220, Sigma).

### Quantitative real-time PCR

TRIzol was used for RNA extraction, and HiScript® II One Step qRT-PCR SYBR® Green KIT (Vazyme) was harnessed to perform real-time PCR. The mRNA levels of target genes were normalized to GAPDH.

The primers (5’−3’) used were:

**Table:** 

Primers	Sequences
IFN-β-s:	TTGTTGAGAACCTCCTGGCT,
IFN-β-as:	TGACTATGGTCCAGGCACAG.
GAPDH-s:	ATGACATCAAGAAGGTGGTG,
GAPDH-as:	CATACCAGGAAATGAGCTTG.
RIG-I-s:	TGTGCTCCTACAGGTTGTGGA,
RIG-I-as:	CACTGGGATCTGATTCGCAAAA.
MDA5-s:	TCACAAGTTGATGGTCCTCAAGT,
MDA5-as:	CTGATGAGTTATTCTCCATGCCC.
VSV P-s:	GTGACGGACGAATGTCTCATAA,
VSV P-as:	TTTGACTCTCGCCTGATTGTAC.
VSV antigenome-s:	GTGACGGACGAATGTCTCATAA,
VSV antigenome-as:	TGATGAATGGATTGGGATAACA.
IL6-s:	GTGTGAAAGCAGCAAAGAGGC,
IL6-as:	CTGGAGGTACTCTAGGTATAC.
IL1β-s:	TCCAGGGACAGGATATGGAG,
IL1β-as:	TCTTTCAACACGCAGGACAG.
TNFα-s:	AGGCGCTCCCCAAGAAGACA,
TNFα-as:	AAGTGCAGCAGGCAGAAGAG.

### Determination of viral titre (TCID50)

Virus titre was determined by TCID50 via endpoint dilution assay. Briefly, Vero cells were grown in a 96-well culture plate, and the medium was discarded when a single cell layer was formed. The virus preparation was diluted in incremental 1:10 fashion with DMEM containing 2% FBS, and 12 replicates of each dilution were added to the Vero cells at a volume of 100 μl per well. The plates were continuously observed until cytopathic effect (CPE) no longer increased in the infected cells. Virus titres were calculated based on the Spearman & Kärber algorithm [34].

### shRNA gene knockdown

The shRNA sequences against negative control (NC) and CDK5 were synthesized by GenePharma (China), and then cloned into the LV−2 vector (pGLVU6/Puro). The viruses were packaged by co-transfecting LV−2 vectors and packing plasmids into HEK293T cells. Upon maturation, the filtered virus suspension with 0.45 μm filterable membrane was added to the cultured cell lines, and puromycin (1 μg/ml) was added to establish stable cell lines. The sequences used for NC and CDK5 were synthesized as follows: TTCTCCGAACGTGTCACGT for NC and CAGAACCTTCTGAAGTGTAAC for CDK5.

### Immunoprecipitation and immunoblotting

Immunoprecipitation and immunoblotting were conducted as previously described [35]. Briefly, cell lysate was extracted from HEK293T cells using NETN buffer containing protease inhibitor (Roche). Anti-Flag or anti-GFP beads were pre-balanced and mixed with the lysate for incubation overnight at 4 °C. For immunoblotting, the protein samples were separated through SDS-PAGE. After Western transfer, the membrane was blocked with 5% milk, and then probed with the indicated antibodies. The immunoblots were visualized using the ImageQuant LAS 4000 system. Quantitate analysis was finished by ImageJ software, and the data are displayed as means ± SE of three independent experiments.

### ELISA

The expression of IFN-β in the cell culture supernatants was measured using an ELISA kit (Proteintech) according to the kit’s instructions.

### Chemical cross-linking

Chemical cross-linking was performed in 293T cells according to the manufacturer’s instructions. Briefly, the cells were washed with PBS and resuspended in PBS containing 0.1 mM of the cross-linking reagent suberic acid bis (N-hydroxysuccinimide ester; BS3, Sigma) for 30 minutes. Tris-HCl (pH 8.0) was then added to a final concentration of 50 mM for 10 minutes to terminate the reaction. After quenching, the samples were processed using the immunoblotting protocol.

### Quantification and statistical analysis

Two-tailed Student’s t-test was performed using the GraphPad Prism software. The statistical significance of each data set is shown in each figure. The results of all statistical analyses were presented as mean ± SE.

## Results

### CDK5 suppresses the production of IFN-β

**Figure 1.**
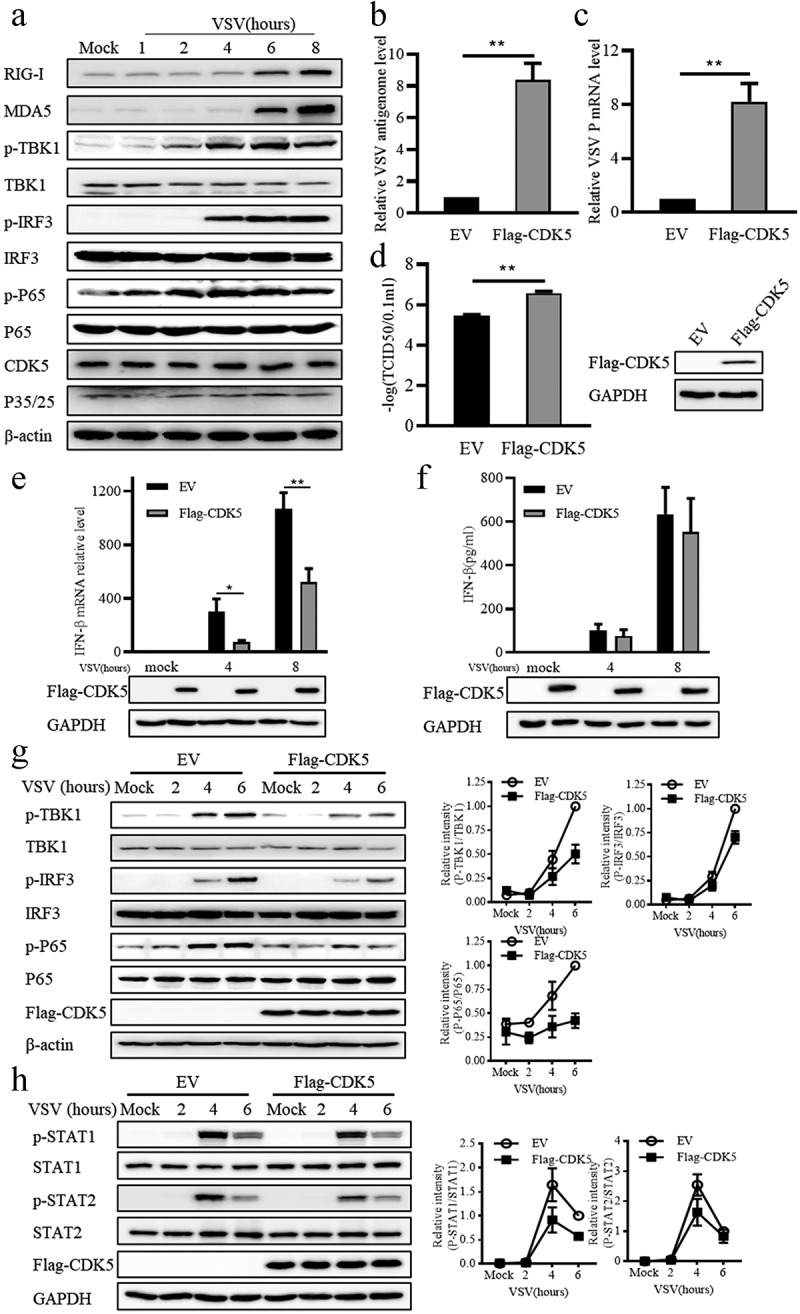
Cyclin dependent kinase 5 (CDK5) suppresses the production of IFN-β.

Note: A. A549 cells were stimulated with VSV-GFP (MOI = 0.1) for 1, 2, 4, 6 or 8 hours, and the phosphorylated or total proteins were detected with specific antibodies.

To identify the potential regulatory function of CDK5 in antiviral immune response, *vesicular stomatitis virus* (VSV) was utilized to detect the changes in the protein levels of CDK5 during infection. Consistently with previous reports [7–9], the results of Western blotting showed that the expression of RIG-I and MDA5 increased over time following VSV infection (Figure 1a). Moreover, as the downstream proteins of RIG-I and MDA5, TBK1, IRF3 and P65 demonstrated enhanced phosphorylation after VSV infection (Figure 1a), while CDK5 expression showed no obvious changes during VSV infection (Figure 1a). Surprisingly, overexpression of CDK5 promoted VSV replication and transcription in the collected cells (Figures 1b,c) and resulted in higher viral titres in the supernatant (Figure 1d), which might be attributed to the low expression of IFN-β induced by CDK5 overexpression (Figure 1e). Consistently, CDK5 overexpression led to a weaker induction of IFN-β protein expression by VSV from the culture supernatant compared to the control (Figure 1f). To ascertain the reason for the decreased transcription of IFN-β, we next detected its upstream signalling proteins. The results of Western blotting indicated that CDK5 overexpression obviously reduced the phosphorylation levels of TBK1, IRF3 and P65, but not their protein levels (Figure 1g). Considering the vital roles of STAT1 and STAT2 in type I IFNs-mediated interferon-stimulated genes (ISGs) expression, we found that CDK5 overexpression obviously attenuated the VSV-induced phosphorylation of STAT1 and STAT2 without affecting their protein levels (Figure 1h). These results demonstrate that CDK5 can enhance VSV infection by negatively regulating IFN-β production in A549 cells.

B-C. After transfection with empty vector (EV) or Flag-CDK5, A549 cells were treated for 10 hours with VSV-GFP (MOI = 0.1), followed by qRT-PCR analysis for detecting the RNA levels of viral genome (B) and the transcription level of VSV P protein (C).

D. TCID50 of VSV in the supernatant of A549 cells overexpressing Flag-CDK5 or EV was measured after infection with VSV-GFP (MOI = 0.1) for 10 hours.

E. EV or Flag-CDK5 was overexpressed in A549 cells, followed by infection with VSV-GFP (MOI = 0.1) for 4 or 8 hours, qRT-PCR analysis (upper panels) and immunoblotting assay (lower panels).

F. A549 cells overexpressing Flag-CDK5 or EV were stimulated with VSV-GFP (MOI = 0.1) for 4 or 8 hours, and IFN-β in supernatant was quantified using the ELISA kit. Lower panels represent immunoblotting assays.

G-H. Left panel: A549 cells overexpressing Flag-CDK5 or EV were treated with VSV-GFP (MOI = 0.1) for 4 or 8 hours, and the changes in protein phosphorylation were detected by Western blotting with specific antibodies. Right panel: Quantitative analysis of the Western blotting results. The relative abundances of P-TBK1, P-IRF3 or P-P65 were normalized to that of total TBK1, IRF3 or P65 (G), and the relative abundances of P-STAT1 or P-STAT2 were normalized to that of total STAT1 or STAT2 (H). All graph values are presented as means ± SE, *n* = 3. *P < 0.05, **P < 0.01.

### CDK5 deficiency promotes the production of IFN-β

**Figure 2.**
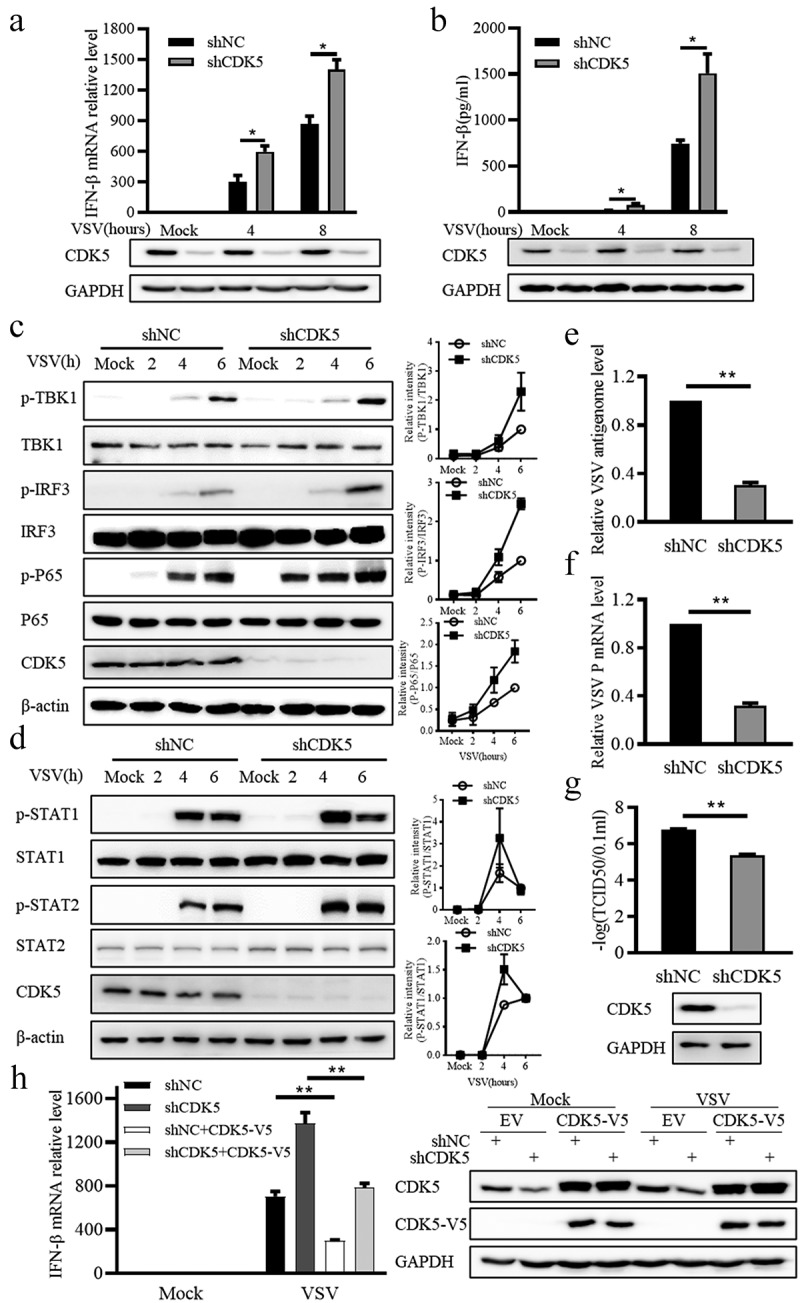
CDK5 deficiency promotes IFN-β production.

Note: A. The mRNA expression of IFN-β in A549 cells that stably expressing short hairpin RNA specific to CDK5 (shCDK5) or negative control (shNC). After infection with VSV-GFP (MOI = 0.1) for 4 or 8 hours, qRT-PCR analysis (upper panels) and immunoblotting assay (lower panels) were conducted.

To investigate whether endogenous CDK5 participates in antiviral immune response, CDK5 knockdown A549 cell lines were constructed with short hairpin RNA lentivirus. After VSV infection, the transcription level of IFN-β was markedly increased in CDK5 knockdown A549 cells (Figure 2a). This was accompanied by a higher level of IFN-β protein in the culture supernatant, as analysed by ELISA (Figure 2b). As upstream signalling molecules of IFN-β production, TBK1, IRF3 and P65 displayed higher phosphorylation in CDK5 knockdown cells after infection with VSV (Figure 2c). In addition, the phosphorylation levels of STAT1/2, the downstream signalling proteins of type I interferon, were markedly enhanced in CDK5-deficient cells (Figure 2d). Subsequently, virus propagation in CDK5-deficient A549 cells was significantly decreased (Figures 2e–g). When rescuing with full-length CDK5 that is resistant to knockdown, we observed a decrease in IFN-β transcription to a level similar to that in wild-type cells (Figure 2h). The above results support the consequence that the increased production in IFN-β during VSV infection may be due to CDK5 deficiency.

B. shNC and shCDK5 A549 cells were stimulated with VSV-GFP (MOI = 0.1) for 4 or 8 hours, and IFN-β in supernatant was quantified using the ELISA kit.

C-D. Left panel: shNC and shCDK5 A549 cells were treated with VSV-GFP (MOI = 0.1) for 2, 4 or 6 hours, the changes in protein phosphorylation were detected by Western blotting with specific antibodies. Right panel: Quantitative analysis of the Western blotting results. The relative abundances of P-TBK1, P-IRF3 or P-P65 were normalized to that of total TBK1, IRF3 or P65 (G), and the relative abundances of P-STAT1 or P-STAT2 were normalized to that of total STAT1 or STAT2 (H).

E-F. shNC and shCDK5 A549 cells were treated with VSV-GFP (MOI = 0.1) for 10 hours, followed by qRT-PCR analysis for detecting the RNA levels of viral genome (E) and the transcription level of VSV P protein (F).

G. TCID50 of VSV in the supernatant of shNC and shCDK5 A549 cells was measured after infection with VSV-GFP (MOI = 0.1) for 10 hours.

H. After stable knockdown of CDK5, A549 cells were transfected with V5-tagged CDK5 resistant plasmid, followed by 8 hours of VSV-GFP (MOI = 0.1) infection. The mRNA expression of IFN-β was quantified by qRT-PCR after RNA extraction. Right panels represent immunoblotting assays.

All graph values are presented as means ± SE, *n* = 3. *P < 0.05, **P < 0.01.

### The regulation of CDK5 in IFN-β production is independent of its kinase activity

**Figure 3.**
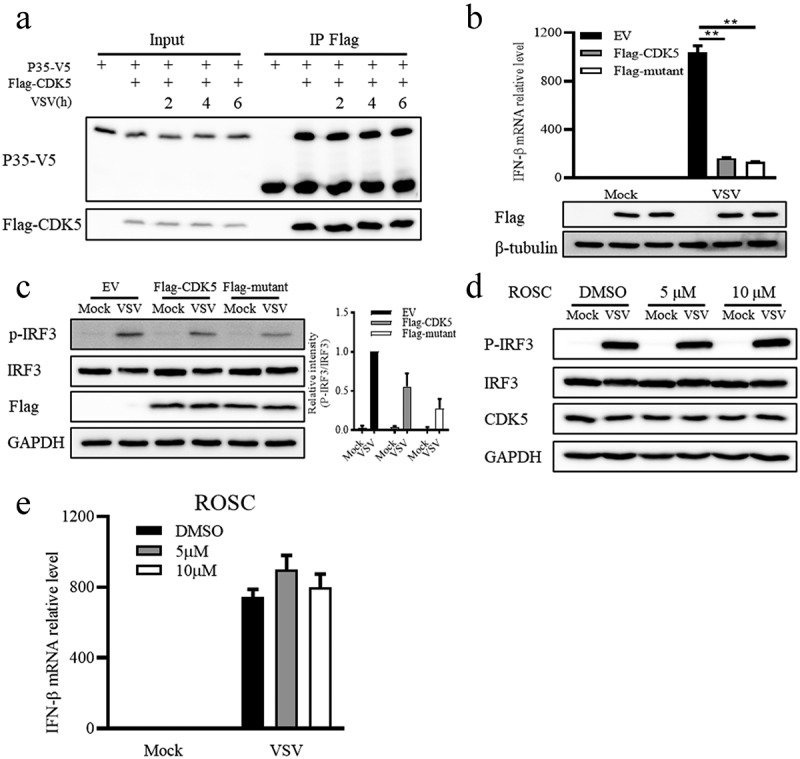
The regulation of CDK5 in IFN-β production is independent of its kinase activity.

Note: A. Flag-CDK5 was co-transfected with P35-V5 into 293T cells. After infection with VSV-GFP for 2, 4 or 6 hours, the cells were lysed and anti-Flag beads were used for immunoprecipitation.

As a kinase, CDK5 can be activated through binding to its activator P35 [36]. Moreover, CDK5 can be hyper-activated via binding to a soluble 25 kDa form (P25), the cleaved form of P35 by calpain under stress stimulation [37]. Therefore, the association of CDK5 with P35 and the occurrence of P25 can be recognized as the change in the kinase activity of CDK5. After VSV infection for the indicated hours, the interaction between CDK5 and P35 remained unaffected (Figure 3a). Moreover, P25 was not observed at the indicated hours of VSV infection (Figure 1a). Overexpression of CDK5 kinase inactive mutant (K33A/E51A/D144A) exhibited a similar effect as wild type CDK5 on decreasing the transcription of IFN-β induced by VSV (Figure 3b). Subsequently, the phosphorylation of IRF3 stimulated by VSV infection also demonstrated the similar extent of suppression in wild type CDK5 or kinase inactive mutant overexpressing cells (Figure 3c). Consistently, treatment with Roscovitine, a small molecule inhibitor for the kinase activity of CDK5, showed no effects on VSV-induced IRF3 phosphorylation or IFN-β transcription compared with control (Figures 3d,e). Therefore, we concluded that CDK5 inhibited the IFN-β signalling pathways induced by VSV infection, in a manner that was not dependent on its kinase activity.

B. After 8 hours of VSV-GFP (MOI = 0.1) infection, the mRNA expression of IFN-β in A549 cells overexpressing Flag-CDK5, Flag-CDK5 kinase inactive mutant (Flag-mutant) or EV was analysed by qRT-PCR assay

C. Left panel: Flag-CDK5, Flag-mutant or EV plasmids were transfected into A549 cells, followed by 8-hour infection with VSV-GFP (MOI = 0.1). Western blotting was performed to determine the phosphorylated or total protein of IRF3. Right panel: Quantitative analysis of the relative abundances of P-IRF3 normalized to that of total IRF3.

D-E. A549 cells were pre-treated with CDK5 kinase inhibitor Roscovitine (ROSC) for 30 minutes, followed by VSV-GFP (MOI = 0.1) infection for 8 hours with ROSC. Western blotting was performed to determine the phosphorylated or total protein of IRF3 (D), and IFN-β expression was analysed by qRT-PCR assay (E).

All graph values are presented as means ± SE, *n* = 3. *P < 0.05, **P < 0.01.

### CDK5 attenuates the transcription of RIG-I and MDA5

**Figure 4.**
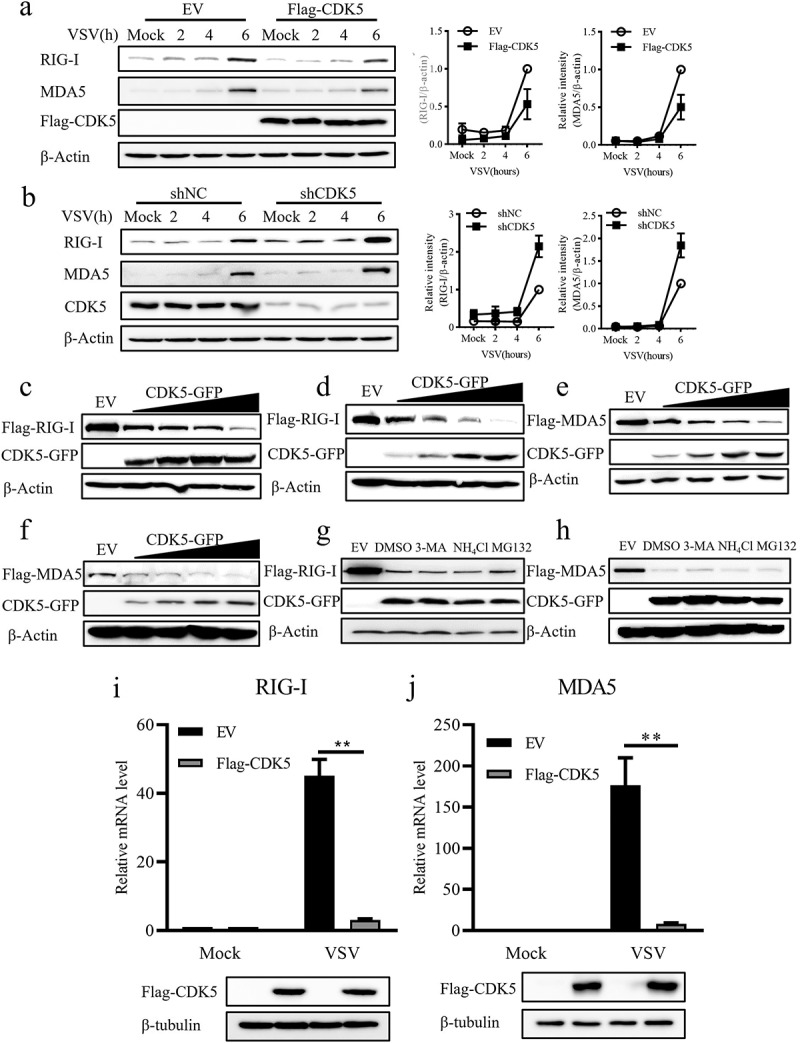
CDK5 attenuates the expression of retinoic acid-inducible gene-I (RIG-I) and melanoma differentiation-associated protein 5 (MDA5).

Note: A-B. Left panel: The expression levels of RIG-I and MDA5 were detected in A549 cells with EV and Flag-CDK5 overexpression (A) or NC and CDK5 knockdown (B) after VSV-GFP (MOI = 0.1) infection for 2, 4 or 6 hours. Right panel: Quantitative analysis of the relative abundances of RIG-I and MDA5 normalized to that of β-Actin in CDK5 overexpressing cells (A) or in CDK5-deficient cells (B).

As CDK5 was found to suppress the activation of TBK1 and IRF3 stimulated by VSV, we sought to investigate whether CDK5 could also impact RIG-I and MDA5, which are known to be upstream signalling proteins of TBK1 and IRF3. Immunoblotting results indicated that the decreased production of RIG-I and MDA5 was driven by CDK5 overexpression during VSV infection (Figure 4a), whereas CDK5 deficiency promoted their expression upon VSV infection (Figure 4b). Furthermore, the reduction in RIG-I and MDA5 induced by CDK5 was found to be dose-dependent in both 293T and A549 cell lines (Figure 4c–f). However, the decreased expression RIG-I and MDA5 could not be blocked by the autophagy inhibitor 3-Methyladenine (3-MA), lysosome inhibitor NH_4_Cl or ubiquitin proteasome inhibitor MG132 (Figure 4g, h), which indicated that this reduction induced by CDK5 might not be attributed to autophagy- or proteasome-mediated protein degradation pathways. Additionally, the data showed that the decreases in RIG-I and MDA5 levels caused by CDK5 overexpression during VSV infection was due to a reduction in their mRNA levels (Figures 4i, j).

C-D. Flag-RIG-I was co-transfected with increasing doses of CDK5-GFP into HEK293T cells (C) or A549 cells (D). After 24 hours of transfection, the expression levels of Flag-RIG-I and CDK5-GFP were determined by Western blotting.

E-F. Flag-MDA5 was co-transfected with increasing doses of CDK5-GFP into HEK293T cells (E) or A549 cells (F). After 24 hours of transfection, the protein levels of Flag-MDA5 and CDK5-GFP were detected by Western blotting.

G. HEK293T cells were transfected with Flag-RIG-I and EV or CDK5-GFP for 24 hours, followed by treatment with 3-MA (10 mM), NH_4_Cl (10 mM) or MG132 (20 μM) for 8 hours. The expression levels of Flag-RIG-I and CDK5-GFP were analysed by Western blotting.

H. HEK293T cells were co-transfected Flag-MDA5 with EV or CDK5-GFP for 24 hours, followed by treatment with 3-MA (10 mM), NH_4_Cl (10 mM) or MG132 (20 μM) for 8 hours. Western blotting demonstrated the expression levels of Flag-MDA5 and CDK5-GFP.

I-J. A549 cells overexpressing Flag-CDK5 or EV were infected with VSV-GFP (MOI = 0.1) for 8 hours. Total RNA was extracted, and the mRNA expression levels of RIG-I (I) and MDA5 (J) were analysed by qRT-PCR assays. Lower panels represent immunoblotting assays.

All graph values are presented as means ± SE, *n* = 3. **P < 0.01.

### CDK5 is associated with MyD88 and suppresses TLRs-MyD88-mediated signalling pathways

**Figure 5.**
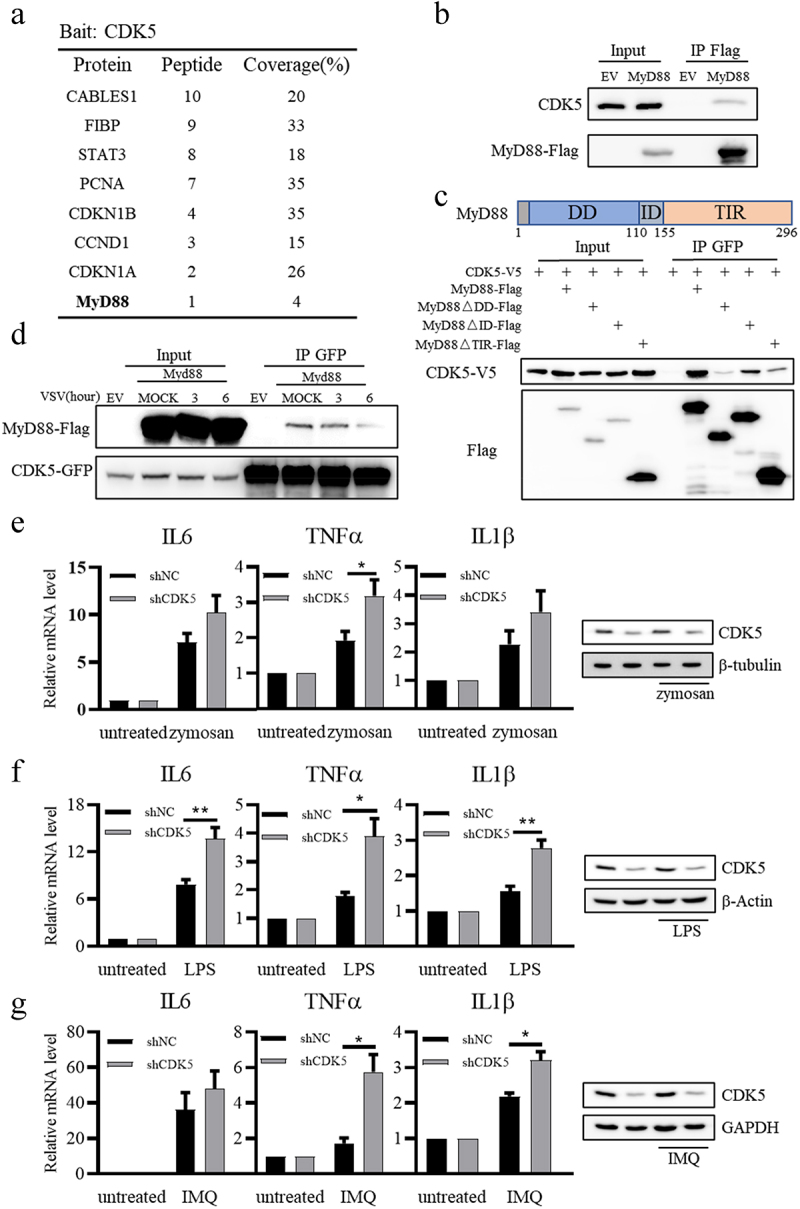
CDK5 is associated with myeloid differentiation primary response gene 88 (MyD88) and suppresses TLR-MyD88-mediated signalling pathways.

Note: A. Identification of CDK5-associated protein via mass spectrometry.

To further elucidate the mechanism by which CDK5 regulates the expression of IFN-β, we purified Flag-CDK5 from A549 cells and analysed the CDK5-associated proteins via mass spectrometry. As shown in Figure 5a, several known CDK5-associated proteins, such as CABLES1, FIBP and STAT3, were identified from the mass spectrometry data [38–40]. Interestingly, MyD88, which is a key adaptor for almost all TLR-dependent signal transduction pathways except TLR3 [10], was also identified among the purified CDK5-associated proteins (Figure 5a). Jennifer M. Lund et al. [41] have reported that VSV can be recognized by TLR7, and MyD88 is required for VSV recognition. It is known that the TLRs-MyD88-IRF7 signalling pathway can induce the production of IFNs, and exposure to IFNs can lead to enhanced RLR signalling [7–9,14,15]. Therefore, we aimed to clarify the role of CDK5 in regulating TLR-MyD88 pathway. Our results showed that CDK5 could interact with MyD88 (Figure 5b), and their interaction was mediated by the TIR and DD domains of MyD88 (Figure 5c). Furthermore, the interaction of CDK5 and MyD88 was attenuated following VSV infection (Figure 5d), suggesting that this interaction was fine-tuned during VSV infection. Considering the broad impact of MyD88 on TLR signalling, we aimed to investigate whether endogenous CDK5 could regulate other MyD88-dependent TLR signalling pathways in A549 cells. As the direct downstream kinase of MyD88, IRAK4 can be phosphorylated after binding to MyD88 and become activated. In response to zymosan (a ligand for TLR2), lipopolysaccharide (a ligand for TLR4), or imiquimod (a ligand for TLR7), the phosphorylation of IRAK4 was boosted in CDK5-deficient A549 cells (Figure S1), while the protein level of CDK5 was not affected by these compounds’ treatments (Figure S2). Besides, CDK5 deficiency in A549 cells increased the transcription levels of IL6, TNFα, and IL1β genes when induced by zymosan, lipopolysaccharide, or imiquimod (Figures 5e–g). Collectively, these data indicate that CDK5 can regulate MyD88-dependent TLR signalling pathway.

B. HEK293T cells were transfected with MyD88-Flag, and MyD88-Flag was pulled down from lysate by anti-Flag beads for Western blotting analysis.

C. Domain mapping of MyD88 and CDK5 interaction was performed by transfecting CDK5-V5 with different domain of MyD88-Flag into HEK293T cells. Anti-Flag beads were mixed into cell lysates for Western blotting analysis.

D. HEK293T cells overexpressing CDK5-GFP and EV or Myd88-Flag plasmids were infected with VSV-GFP (MOI = 0.1) for 3 or 6 hours. Cells were lysed, and CDK5-GFP was pulled down by anti-GFP beads for Western blotting analysis.

E-G. Effects of CDK5 on TLR2 (E), TLR4 (F) and TLR7 (G) signalling pathways. shNC and shCDK5 A549 cells were stimulated with zymosan (100 ng/ml) (E), lipopolysaccharide (LPS) (100 ng/ml) (F) or imiquimod (IMQ) (50 ng/ml) (G) for 2 hours, followed by qRT-PCR analysis (left panels) and immunoblotting assay (right panels).

All graph values are presented as means ± SE, *n* ≥ 3. *P < 0.05, **P < 0.01.

### CDK5 attenuates the formation of MyD88 homodimers independent of its kinase activity

**Figure 6.**
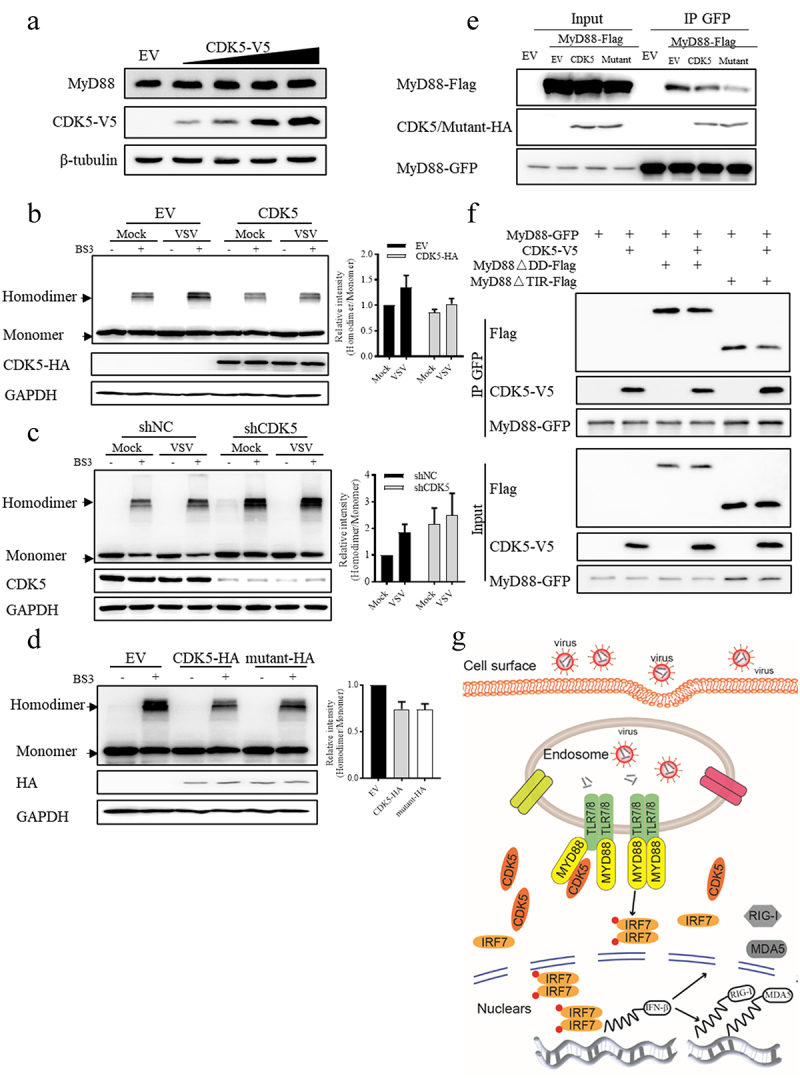
CDK5 negatively regulates the formation of MyD88 homodimers independent of its kinase activity.

Note: A. Endogenous expression patterns of MyD88 in A549 cells transfected with increasing doses of CDK5.

To explore the mechanism by which CDK5 regulates MyD88-dependent TLR signalling pathway, we first examined the protein level of MyD88 after CDK5 expression. Unexpectedly, we found that the protein level of MyD88 was not affected by CDK5 overexpression, as shown by the results of Western blotting analysis (Figure 6a). Upon pathogenic infection, MyD88 undergoes aggregation to form a dimer, which is crucial for its activation and downstream signal transduction in the TLR-MyD88 signalling pathway [12,13,42]. Therefore, we investigated the effect of CDK5 on MyD88 homodimer formation. We found that the overexpression of CDK5 led to a decrease in the formation of MyD88 homodimers (Figure 6b). Conversely, when CDK5 was knocked down, we observed an increase in the formation of MyD88 homodimers (Figure 6c). Consistent with the results obtained from wild-type CDK5, we found that the overexpression of its kinase inactive mutant (K33A/E51A/D144A) also attenuated the formation of MyD88 homodimers (Figure 6d), which might be attributed to the decreased self-association of MyD88 induced by CDK5 overexpression (Figure 6e). As the self-association of MyD88 is mediated by its DD and TIR domain, we attempted to investigate how CDK5 affected the self-association of MyD88. The results showed that CDK5 could decrease both DD domain- and TIR domain-mediated self-association of MyD88 (Figure 6f). Collectively, these results imply that CDK5 can decrease the formation of MyD88 homodimers independent of its kinase activity.

B. CDK5 attenuated the formation of MyD88 homodimers. MyD88-GFP was co-transfected with EV or CDK5-HA into HEK293T for 24 hours. After 3 hours of VSV-GFP (MOI = 0.1) infection, the cells were crosslinked by 0.1 mM BS3 for 30 minutes. The cell lysates were prepared after terminating the crosslink reaction for immunoblotting analysis. The expression of CDK5-HA was detected from the sample collected before cross-linkage. The right panel displayed the quantitative analysis of the relative abundances of MyD88 homodimers, which were crosslinked with BS3 and normalized to the monomer formation of MyD88.

C. Left panel: CDK5 deficiency promoted the formation of MyD88 homodimer. shNC and shCDK5 HEK293T cells were transfected with MyD88-GFP. The cells were then crosslinked by 0.1 mM BS3 for 30 minutes after 3 hours of VSV-GFP (MOI = 0.1) infection. The cell lysates were prepared after terminating the crosslink reaction for immunoblotting analysis. The right panel displayed the quantitative analysis of the relative abundances of MyD88 homodimers, which were crosslinked with BS3 and normalized to the monomer formation of MyD88.

D. Left panel: The formation of MyD88 homodimers was attenuated by CDK5 kinase inactive mutant. MyD88-GFP was co-transfected with EV, CDK5-HA WT (CDK5-HA) or CDK5-HA kinase dead mutant (mutant-HA) into HEK293T for 24 hours. The cells were crosslinked for 30 minutes with 0.1 mM BS3. The cell lysates were prepared after terminating the crosslink reaction for immunoblotting analysis. The right panel displays the quantitative analysis of the relative abundances of MyD88 homodimers, which were crosslinked with BS3 and normalized to the monomer formation of MyD88.

E. The self-interaction of MyD88 was attenuated by CDK5 or its kinase inactive mutant. After transfection with the indicated plasmids for 24 hours, MyD88-GFP in HEK293T cells was immunoprecipitated by anti-GFP beads for Western blotting analysis.

F. CDK5 suppressed the self-association of MyD88 by disturbing both the DD and TIR domains of MyD88. After transfection with the indicated plasmids for 24 hours, MyD88-GFP in HEK293T cells was immunoprecipitated by anti-GFP beads for Western blotting analysis.

G. Model of CDK5 in the regulation of IFN-β production triggered by RNA virus via TLR-MyD88 pathway.

All graph values are presented as means ± SE, *n* = 3.

## Discussion

As part of the PRRs in the innate immune system, TLRs have been demonstrated to play a crucial role in enabling the host to resist pathogen attacks. In the present study, we uncovered that CDK5 exhibited an inhibitory effect on the production of IFN-β induced by VSV infection. Mechanistically, CDK5 was associated with MyD88 and hindered the formation of MyD88 homodimers, thus resulting in the decreased production of IFN-β. As a consequence, the expression levels of RIG-I and MDA5, which are induced by IFNs, were also dampened, resulting in a further reduction in the production of IFNs (Figure 6g).

Previously, CDK5 was believed to be a kinase that exclusively functions within the nervous system, despite its widespread expression. However, with a growing body of evidence, CDK5 has been demonstrated to participate in a wide range of functions beyond the nervous system. Several reports have shown that inhibition of CDK5 could suppress inflammation in immune cells. In inflammation macrophages, the activated CDK5 decreases IL−10 production by inhibiting the phosphorylation and activation of mitogen activated protein kinases (MAPKs) [43]. Conversely, downregulation of CDK5 expression or inhibition of its kinase activity has been shown to enhance the anti-inflammatory effect [44]. Besides, CDK5 activity has been reported to be necessary for T cell activation and maintaining its proper distribution [45]. In neutrophils, CDK5 can regulate the secretion of cytokines during infection or injury by phosphorylating vimentin [46].

Unlike the pro-inflammatory role of CDK5 in immune cells, our study revealed that CDK5 exerts a negative regulatory effect on the antiviral immune response during VSV infection in A549 cells, and its kinase activity does not appear to be involved in the antiviral immune response. In contrast to studies on macrophages [43,44] and microglia [47], which demonstrated that CDK5 or P35 expression was upregulated upon inflammatory stimuli, we observed no significant changes in the protein level of CDK5 or P35 in A549 cells after VSV infection or treatment with zymosan, lipopolysaccharide, or imiquimod. We hypothesize that the kinase-independent function of CDK5 in antiviral immunity results in the consistent level of P35, or the lack of P25 production during VSV infection, as demonstrated in Figure 1a. In addition, CDK5 exhibits distinct functions in dopamine neurons [48]. Changes in CDK5 activity can impair autophagic flux, resulting in hyperactive innate immunity. However, in our study, treatment with a CDK5 kinase inhibitor had no impact on the antiviral immunity in A549 cells. In addition, our results showed that overexpression of CDK5 in raw 264.7 macrophage cell line increased the transcription of IFN-β upon VSV infection (Figure S3), suggesting that CDK5 may positively regulate antiviral immunity in mouse immune cells, which was inconsistent with that from A549 cells. The diverse functional models of CDK5 in different cell types indicate that CDK5 may function in a cell type-specific manner. Taken together, our findings suggest that CDK5 serves as a distinct factor in regulating the activation of immune responses between immune cells and non-immune cells.

Appropriate inflammatory responses are beneficial in response to infections or cellular damage, but chronic inflammatory stimulation can lead to the escape of tumour cells from immunosurveillance due to the development of cancer cells’ resistance to inflammation [49]. As a key adaptor protein in most TLRs, it is widely acknowledged that MyD88 can function as a double-edged sword, capable of both adaptable and injurious inflammation [21]. Hence, the activation of MyD88 must be finely controlled. MyD88 can be ubiquitinated by E3 ubiquitin ligases such as neuregulin receptor degradation protein 1 (Nrdp1), speckle-type BTB – POZ protein (SPOP), or Smad ubiquitin regulator factor 1 (Smurf1), and the ubiquitinated MyD88 is then subject to degradation [16,17,[50]]. In addition, MyD88 can be dephosphorylated by protein phosphatase 2A catalytic subunit (PP2Ac), Src homology−2-containing protein tyrosine phosphatase−2 (SHP2), or phosphorylated by spleen tyrosine kinase (SYK), eventually affecting MyD88 activity [19,21,[51]]. More importantly, the activity of MyD88 can be regulated by affecting its self-association [13]. In this study, we demonstrated that CDK5 can negatively regulate MyD88-dependent signalling pathway by disrupting the self-association of MyD88, while the kinase activity of CDK5 does not appear to play a role in this process. CDK5 interacts with the DD and TIR domains of MyD88, thus disrupting the formation of MyD88 homodimers.

CDK5 or its activators P35 and P39 are reported to be hyper-expressed in many cancers, such as breast cancers, head and neck cancers, colon cancers, lung cancers, prostate cancers, yolk sac tumour, seminoma cancers [25,52–54], while hypo-expressed in brain and central nervous system (CNS) cancers, leukaemia cancers and liver cancers [27]. Deficiency of CDK5 or treatment with Roscovitine results in the inhibited proliferation of lung cancer cells [27,[55]]. Furthermore, Roscovitine treatment has been shown to enhance the cytotoxic activity of camptothecin (CPT) in drug-resistant small cell lung cancer (SCLC) cell lines, which may improve the anticancer therapy in SCLC [56]. In addition, various CDK5 inhibitors have been used in preclinical trials for several types of tumours [54]. Given that the expression of CDK5 is increased in all lung malignancies, and that higher expression of CDK5 in patients with lung cancer is associated with a poorer prognosis [27,57,58], CDK5 inhibitors could be used for treating both NSCLC and SCLC. It is generally acknowledged that type I interferons exert the antitumor ability and have been applied for antitumor therapy [59–62]. Our data revealed a kinase-independent function of CDK5 in regulating IFN-β in A549 cells during VSV infection, this finding also suggests a potential therapy for cancers by targeting the protein level of CDK5.

Oncolytic virotherapy (OV) is an emerging approach for cancer therapy that utilizes viruses to selectively destroy tumour cells while sparing healthy cells [63]. VSV is considered a promising OV that can selectively infect cancer cells exhibiting impaired antiviral responses induced by type I IFN [64]. Our data suggest that CDK5 has the potential to suppress IFN-β during VSV infection, and CDK5 expression is higher in most tumour cells than in normal cells, consistent with our conclusion that CDK5 enhances VSV infection by negatively regulating IFN-β production. This finding may aid in the development of a more effective oncolytic virus by manipulating VSV to better target cancer cells.

In summary, our study reveals that CDK5 functions as a suppressor of TLR-induced type I IFN expression by disrupting the self-association of MyD88. This highlights a previously unknown role of CDK5 in regulating the antiviral immune response and provides insights into the potential application of CDK5 as a therapeutic target in immune-related diseases. Due to the essential biological roles of both IFNs and TLRs in antiviral responses, it is crucial to regulate their activities precisely. Hence, precise control of TLR activity through CDK5 may hold significant promise as an intervention for viral diseases.

## Supplementary Material

Supplemental Material

## Data Availability

The authors confirm that the data supporting the findings of this study are available within the article and its supplementary materialshttps://doi.org/10.6084/m9.figshare.23512494.

## References

[1] Akira S, Uematsu S, Takeuchi O. Pathogen recognition and innate immunity. Cell. 2006;124(4):783–17. Epub 2006/02/25 PubMed PMID: 16497588. doi: 10.1016/j.cell.2006.02.015 16497588

[2] Yoneyama M, Fujita T. RNA recognition and signal transduction by RIG-I-like receptors. Immunol Rev. 2009;227(1):54–65. Epub 2009/01/06 PubMed PMID: 19120475. doi:10.1111/j.1600-065X.2008.00727.x. 19120475

[3] Seth RB, Sun L, Ea CK, et al. Identification and characterization of MAVS, a mitochondrial antiviral signaling protein that activates NF-kappaB and IRF 3. Cell. 2005;122(5):669–682. Epub 2005/08/30 PubMed PMID: 16125763. doi: 10.1016/j.cell.2005.08.012 16125763

[4] Kawai T, Takahashi K, Sato S, et al. IPS-1, an adaptor triggering RIG-I- and Mda5-mediated type I interferon induction. Nat Immunol. 2005;6(10):981–988. Epub 2005/08/30 PubMed PMID: 16127453. doi:10.1038/ni1243. 16127453

[5] Xu LG, Wang YY, Han KJ, et al. VISA is an adapter protein required for virus-triggered IFN-beta signaling. Mol Cell. 2005;19(6):727–740. Epub 2005/09/13 PubMed PMID: 16153868. doi:10.1016/j.molcel.2005.08.014. 16153868

[6] Fitzgerald KA, McWhirter SM, Faia KL, et al. Ikkepsilon and TBK1 are essential components of the IRF3 signaling pathway. Nat Immunol. 2003;4(5):491–496. Epub 2003/04/15 PubMed PMID: 12692549. doi:10.1038/ni921. 12692549

[7] Honda K, Takaoka A, Taniguchi T. Type I interferon [corrected] gene induction by the interferon regulatory factor family of transcription factors. Immunity. 2006;25(3):349–360. Epub 2006/09/19 PubMed PMID: 16979567. doi:10.1016/j.immuni.2006.08.009. 16979567

[8] Kang DC, Gopalkrishnan RV, Wu Q, et al. Mda-5: an interferon-inducible putative RNA helicase with double-stranded RNA-dependent ATPase activity and melanoma growth-suppressive properties. Proc Natl Acad Sci U S A. 2002;99(2):637–642. Epub 2002/01/24 PubMed PMID: 11805321; PubMed Central PMCID: PMCPMC117358. doi: 10.1073/pnas.022637199 11805321 PMC117358

[9] Kang DC, Gopalkrishnan RV, Lin L, et al. Expression analysis and genomic characterization of human melanoma differentiation associated gene-5, mda-5: a novel type I interferon-responsive apoptosis-inducing gene. Oncogene. 2004;23(9):1789–1800. Epub 2003/12/17 PubMed PMID: 14676839. doi:10.1038/sj.onc.1207300. 14676839

[10] Moresco EM, LaVine D, Beutler B. Toll-like receptors. Curr Biol. 2011;21(13):R488–93. Epub 2011/07/12 PubMed PMID: 21741580. doi:10.1016/j.cub.2011.05.039. 21741580

[11] Beutler BA. TLRs and innate immunity. Blood. 2009;113(7):1399–1407. Epub 2008/09/02 PubMed PMID: 18757776; PubMed Central PMCID: PMCPMC2644070. doi:10.1182/blood-2008-07-019307 18757776 PMC2644070

[12] Into T, Inomata M, Niida S, et al. Regulation of MyD88 aggregation and the MyD88-dependent signaling pathway by sequestosome 1 and histone deacetylase 6. J Biol Chem. 2010;285(46):35759–35769. Epub 2010/09/15 PubMed PMID: 20837465; PubMed Central PMCID: PMCPMC2975200. doi:10.1074/jbc.M110.126904. 20837465 PMC2975200

[13] Hu YH, Wang Y, Wang F, et al. SPOP negatively regulates Toll-like receptor-induced inflammation by disrupting MyD88 self-association. Cell Mol Immunol. 2020;18:1708–1717. Epub 2020/04/03 PubMed PMID: 32235916. doi:10.1038/s41423-020-0411-1 32235916 PMC8245473

[14] Honda K, Yanai H, Mizutani T, et al. Role of a transductional-transcriptional processor complex involving MyD88 and IRF-7 in Toll-like receptor signaling. Proc Natl Acad Sci U S A. 2004;101(43):15416–15421. Epub 2004/10/20 PubMed PMID: 15492225; PubMed Central PMCID: PMCPMC523464. doi:10.1073/pnas.0406933101. 15492225 PMC523464

[15] Kawai T, Sato S, Ishii KJ, et al. Interferon-alpha induction through Toll-like receptors involves a direct interaction of IRF7 with MyD88 and TRAF6. Nat Immunol. 2004;5(10):1061–1068. Epub 2004/09/14 PubMed PMID: 15361868. doi:10.1038/ni1118. 15361868

[16] Lee YS, Park JS, Kim JH, et al. Smad6-specific recruitment of Smurf E3 ligases mediates TGF-beta1-induced degradation of MyD88 in TLR4 signalling. Nat Commun. 2011;2:460. Epub 2011/09/08PubMed PMID: 21897371. doi:10.1038/ncomms1469 21897371

[17] Wang C, Chen T, Zhang J, et al. The E3 ubiquitin ligase Nrdp1 ‘preferentially’ promotes TLR-mediated production of type I interferon. Nat Immunol. 2009;10(7):744–752. Epub 2009/06/02 PubMed PMID: 19483718. doi:10.1038/ni.1742. 19483718

[18] Han C, Jin J, Xu S, et al. Integrin CD11b negatively regulates TLR-triggered inflammatory responses by activating Syk and promoting degradation of MyD88 and TRIF via Cbl-b. Nat Immunol. 2010;11(8):734–742. Epub 20100718 PubMed PMID: 20639876. doi:10.1038/ni.1908. 20639876

[19] Gurung P, Fan G, Lukens JR, et al. Tyrosine Kinase SYK licenses MyD88 adaptor protein to instigate IL-1alpha-mediated inflammatory disease. Immunity. 2017;46(4):635–648. Epub 2017/04/16 PubMed PMID: 28410990; PubMed Central PMCID: PMCPMC5501252. doi:10.1016/j.immuni.2017.03.014. 28410990 PMC5501252

[20] Loiarro M, Volpe E, Ruggiero V, et al. Mutational analysis identifies residues crucial for homodimerization of myeloid differentiation factor 88 (MyD88) and for its function in immune cells. J Biol Chem. 2013;288(42):30210–30222. Epub 20130909 PubMed PMID: 24019529; PubMed Central PMCID: PMCPMC3798488. doi:10.1074/jbc.M113.490946. 24019529 PMC3798488

[21] Xie L, Liu C, Wang L, et al. Protein phosphatase 2A catalytic subunit alpha plays a MyD88-dependent, central role in the gene-specific regulation of endotoxin tolerance. Cell Rep. 2013;3(3):678–688. Epub 2013/02/26 PubMed PMID: 23434512; PubMed Central PMCID: PMCPMC4060247. doi:10.1016/j.celrep.2013.01.029. 23434512 PMC4060247

[22] Morgan DO. Principles of CDK regulation. Nature. 1995;374(6518):131–134. Epub 1995/03/09 PubMed PMID: 7877684. doi: 10.1038/374131a0 7877684

[23] Dhavan R, Tsai LH. A decade of CDK5. Nat Rev Mol Cell Biol. 2001;2(10):749–759. Epub 2001/10/05 PubMed PMID: 11584302. doi:10.1038/35096019 11584302

[24] Humbert S, Dhavan R, Tsai L. P39 activates cdk5 in neurons, and is associated with the actin cytoskeleton. J Cell Sci. 2000;113(Pt 6):975–983. Epub 2000/02/22. PubMed PMID: 10683146. doi:10.1242/jcs.113.6.975. 10683146

[25] Zhuang K, Zhang J, Xiong M, et al. CDK5 functions as a tumor promoter in human colorectal cancer via modulating the ERK5-AP-1 axis. Cell Death Dis. 2016;7(10):e2415. Epub 2016/10/14 PubMed PMID: 27735944; PubMed Central PMCID: PMCPMC5133995. doi:10.1038/cddis.2016.333. 27735944 PMC5133995

[26] Zhang X, Zhong T, Dang Y, et al. Aberrant expression of CDK5 infers poor outcomes for nasopharyngeal carcinoma patients. Int J Clin Exp Pathol. 2015;8(7):8066–8074. Epub 2015/09/05. PubMed PMID: 26339373; PubMed Central PMCID: PMCPMC4555701. 26339373 PMC4555701

[27] Zeng J, Xie S, Liu Y, et al. CDK5 functions as a tumor promoter in human lung cancer. J Cancer. 2018;9(21):3950–3961. Epub 2018/11/10 PubMed PMID: 30410599; PubMed Central PMCID: PMCPMC6218768. doi:10.7150/jca.25967. 30410599 PMC6218768

[28] Contreras-Vallejos E, Utreras E, Gonzalez-Billault C. Going out of the brain: non-nervous system physiological and pathological functions of Cdk5. Cell Signal. 2012;24(1):44–52. Epub 2011/09/20PubMed PMID: 21924349. doi:10.1016/j.cellsig.2011.08.022. 21924349

[29] Arif A. Extraneuronal activities and regulatory mechanisms of the atypical cyclin-dependent kinase Cdk5. Biochem Pharmacol. 2012;84(8):985–993. Epub 2012/07/17 PubMed PMID: 22795893. doi:10.1016/j.bcp.2012.06.027. 22795893

[30] Lam E, Choi SH, Pareek TK, et al. Cyclin-dependent kinase 5 represses Foxp3 gene expression and Treg development through specific phosphorylation of Stat3 at Serine 727. Mol Immunol. 2015;67(2 Pt B):317–324. Epub 2015/07/23 PubMed PMID: 26198700; PubMed Central PMCID: PMCPMC4734131. doi:10.1016/j.molimm.2015.06.015. 26198700 PMC4734131

[31] Lam E, Pareek TK, Letterio JJ. Cdk5 controls IL-2 gene expression via repression of the mSin3a-HDAC complex. Cell Cycle. 2015;14(8):1327–1336. Epub 2015/03/19 PubMed PMID: 25785643; PubMed Central PMCID: PMCPMC4614394. doi:10.4161/15384101.2014.987621 25785643 PMC4614394

[32] Pareek TK, Lam E, Zheng XJ, et al. Cyclin-dependent kinase 5 activity is required for T cell activation and induction of experimental autoimmune encephalomyelitis. J Exp Med. 2010;207(11):2507–2519. PubMed PMID: WOS:000285504900019. doi:10.1084/jem.20100876. 20937706 PMC2964575

[33] Zhao YY, Sun XF, Nie XL, et al. COX5B regulates MAVS-mediated antiviral signaling through interaction with ATG5 and repressing ROS production. PLoS Pathog. 2012;8(12). ARTN e1003086 10.1371/journal.ppat.1003086. PubMed PMID: WOS:000312907100038. PMC3534373 23308066

[34] Lei C, Yang J, Hu J, et al. On the calculation of TCID50 for quantitation of virus infectivity. Virol Sin. 2021;36(1):141–144. Epub 2020/05/28 PubMed PMID: 32458296; PubMed Central PMCID: PMCPMC7973348. doi:10.1007/s12250-020-00230-5. 32458296 PMC7973348

[35] Wang JQ, Zhu S, Wang YH, et al. Miro2 supplies a platform for Parkin translocation to damaged mitochondria. Sci Bull. 2019;64(11):730–747. PubMed PMID: WOS:000472947800004. doi:10.1016/j.scib.2019.04.033. 36659543

[36] Tarricone C, Dhavan R, Peng J, et al. Structure and regulation of the CDK5-p25(nck5a) complex. Mol Cell. 2001;8(3):657–669. Epub 2001/10/05 PubMed PMID: 11583627. doi:10.1016/s1097-2765(01)00343-4. 11583627

[37] Patrick GN, Zukerberg L, Nikolic M, et al. Conversion of p35 to p25 deregulates Cdk5 activity and promotes neurodegeneration. Nature. 1999;402(6762):615–622. Epub 1999/12/22 PubMed PMID: 10604467. doi:10.1038/45159. 10604467

[38] Zukerberg LR, Patrick GN, Nikolic M, et al. Cables links Cdk5 and c-Abl and facilitates Cdk5 tyrosine phosphorylation, kinase upregulation, and neurite outgrowth. Neuron. 2000;26(3):633–646. Epub 2000/07/15 PubMed PMID: 10896159. doi:10.1016/s0896-6273(00)81200-3. 10896159

[39] Xu S, Li X, Gong Z, et al. Proteomic analysis of the human cyclin-dependent kinase family reveals a novel CDK5 complex involved in cell growth and migration. Mol & Cell Proteomics. 2014;13(11):2986–3000. Epub 2014/08/07 PubMed PMID: 25096995; PubMed Central PMCID: PMCPMC4223486. doi:10.1074/mcp.M113.036699. PMC4223486 25096995

[40] Lin H, Chen MC, Chiu CY, et al. Cdk5 regulates STAT3 activation and cell proliferation in medullary thyroid carcinoma cells. J Biol Chem. 2007;282(5):2776–2784. Epub 2006/12/06 PubMed PMID: 17145757. doi:10.1074/jbc.M607234200. 17145757

[41] Lund JM, Alexopoulou L, Sato A, et al. Recognition of single-stranded RNA viruses by Toll-like receptor 7. Proc Natl Acad Sci U S A. 2004;101(15):5598–5603. Epub 2004/03/23 PubMed PMID: 15034168; PubMed Central PMCID: PMCPMC397437. doi:10.1073/pnas.0400937101. 15034168 PMC397437

[42] Lin SC, Lo YC, Wu H. Helical assembly in the MyD88-IRAK4-IRAK2 complex in TLR/IL-1R signalling. Nature. 2010;465(7300):885–890. Epub 2010/05/21 PubMed PMID: 20485341; PubMed Central PMCID: PMCPMC2888693. doi:10.1038/nature09121. 20485341 PMC2888693

[43] Na YR, Jung D, Gu GJ, et al. The early synthesis of p35 and activation of CDK5 in LPS-stimulated macrophages suppresses interleukin-10 production. Sci Signal. 2015;8(404):ra121. Epub 2015/11/26 PubMed PMID: 26602020. doi:10.1126/scisignal.aab3156. 26602020

[44] Pfander P, Fidan M, Burret U, et al. Cdk5 deletion enhances the anti-inflammatory potential of GC-Mediated GR activation during inflammation. Front Immunol. 2019;10:1554. Epub 2019/07/30 PubMed PMID: 31354714; PubMed Central PMCID: PMCPMC6635475. doi:10.3389/fimmu.2019.01554 31354714 PMC6635475

[45] Pareek TK, Lam E, Zheng X, et al. Cyclin-dependent kinase 5 activity is required for T cell activation and induction of experimental autoimmune encephalomyelitis. J Exp Med. 2010;207(11):2507–2519. Epub 2010/10/13 PubMed PMID: 20937706; PubMed Central PMCID: PMCPMC2964575. doi:10.1084/jem.20100876. 20937706 PMC2964575

[46] Askew D, Pareek TK, Eid S, et al. Cyclin-dependent kinase 5 activity is required for allogeneic T-cell responses after hematopoietic cell transplantation in mice. Blood. 2017;129(2):246–256. Epub 20161114 PubMed PMID: 28064242; PubMed Central PMCID: PMCPMC5234215. doi:10.1182/blood-2016-05-702738. 28064242 PMC5234215

[47] Wang X., Sun L., Guan S., Yan H., Huang X., Liang M., Zhang R., & Luo, T. Cyclin-dependent kinase 5 inhibitor attenuates lipopolysaccharide-induced neuroinflammation through metabolic reprogramming. Eur J Pharmacol. 2022;929:175118. doi:10.1016/j.ejphar.2022.175118 35787890

[48] Shukla AK, Spurrier J, Kuzina I, et al. Hyperactive Innate immunity causes degeneration of dopamine neurons upon altering activity of Cdk5. Cell Rep. 2019;: . PubMed PMID: 30605670; PubMed Central PMCID: PMCPMC6442473. doi:10.1016/j.celrep.2018.12.025. PMC6442473 30605670

[49] Rakoff-Nahoum, S., & Medzhitov, R. Toll-like receptors and cancer. Nat Rev Cancer. 2009;9(1):57–63. doi: 10.1038/nrc2541 19052556

[50] Guillamot, M. The E3 ubiquitin ligase SPOP controls resolution of systemic inflammation by triggering MYD88 degradation. Nat Immunol. 2019;20(9):1196–1207. doi:10.1038/s41590-019-0454-6 31406379 PMC7376385

[51] Heun Y, Pircher J, Czermak T, et al. Inactivation of the tyrosine phosphatase SHP-2 drives vascular dysfunction in Sepsis. EBioMedicine. 2019;42:120–132. Epub 2019/03/25 PubMed PMID: 30905847; PubMed Central PMCID: PMCPMC6491420. doi:10.1016/j.ebiom.2019.03.034. 30905847 PMC6491420

[52] Liang Q, Li L, Zhang J, et al. CDK5 is essential for TGF-beta1-induced epithelial-mesenchymal transition and breast cancer progression. Sci Rep. 2013;3:2932. Epub 2013/10/15 PubMed PMID: 24121667; PubMed Central PMCID: PMCPMC3796304. doi:10.1038/srep02932 24121667 PMC3796304

[53] Oner M, Lin E, Chen MC, et al. Future aspects of CDK5 in prostate cancer: from pathogenesis to therapeutic implications. Int J Mol Sci. 2019;20(16):Epub 2019/08/10 PubMed PMID: 31395805; PubMed Central PMCID: PMCPMC6720211. doi:10.3390/ijms20163881. PMC6720211 31395805

[54] Gao GB, Sun Y, Fang RD, et al. Post-translational modifications of CDK5 and their biological roles in cancer. Mol Biomed. 2021;2(1):22. Epub 2022/01/11 PubMed PMID: 35006426; PubMed Central PMCID: PMCPMC8607427. doi: 10.1186/s43556-021-00029-0 35006426 PMC8607427

[55] Zeng Y, Liu Q, Wang Y, et al. CDK5 activates hippo signaling to confer resistance to radiation therapy via upregulating TAZ in lung cancer. Int J Radiat Oncol Biol Phys. 2020;108(3):758–769. Epub 2020/05/15 PubMed PMID: 32407930. doi:10.1016/j.ijrobp.2020.05.005. 32407930

[56] Hamilton G, Klameth L, Rath B, et al. Synergism of cyclin-dependent kinase inhibitors with camptothecin derivatives in small cell lung cancer cell lines. Molecules. 2014;19(2):2077–2088. Epub 2014/02/20PubMed PMID: 24549232; PubMed Central PMCID: PMCPMC6271949. doi:10.3390/molecules19022077 24549232 PMC6271949

[57] Prince G, Yang TY, Lin H, et al. Mechanistic insight of cyclin-dependent kinase 5 in modulating lung cancer growth. Chin J Physiol. 2019;62(6):231–240. Epub 2019/12/04 PubMed PMID: 31793458. doi:10.4103/CJP.CJP_67_19. 31793458

[58] Liu JL, Wang XY, Huang BX, et al. Expression of CDK5/p35 in resected patients with non-small cell lung cancer: relation to prognosis. Med Oncol. 2011;28(3):673–678. Epub 2010/04/01 PubMed PMID: 20354813. doi:10.1007/s12032-010-9510-7. 20354813

[59] Zitvogel L, Galluzzi L, Kepp O, et al. Type I interferons in anticancer immunity. Nat Rev Immunol. 2015;15(7):405–414. Epub 2015/06/02 PubMed PMID: 26027717. doi:10.1038/nri3845. 26027717

[60] Yang X, Zhang X, Fu ML, et al. Targeting the tumor microenvironment with interferon-beta bridges innate and adaptive immune responses. Cancer Cell. 2014;25(1):37–48. Epub 2014/01/18 PubMed PMID: 24434209; PubMed Central PMCID: PMCPMC3927846. doi:10.1016/j.ccr.2013.12.004. 24434209 PMC3927846

[61] Vacchelli E, Aranda F, Eggermont A, et al. Trial Watch: tumor-targeting monoclonal antibodies in cancer therapy. Oncoimmunology. 2014;3(1):e27048. Epub 2014/03/08 PubMed PMID: 24605265; PubMed Central PMCID: PMCPMC3937194. doi:10.4161/onci.27048. 24605265 PMC3937194

[62] Chen KS, Reinshagen C, Van Schaik TA, et al. Bifunctional cancer cell-based vaccine concomitantly drives direct tumor killing and antitumor immunity. Sci Transl Med. 2023;15(677):eabo4778. Epub 2023/01/05 PubMed PMID: 36599004; PubMed Central PMCID: PMCPMC10068810. doi:10.1126/scitranslmed.abo4778. 36599004 PMC10068810

[63] Russell SJ, Peng KW, Bell JC. Oncolytic virotherapy. Nat Biotechnol. 2012;30(7):658–670. Epub 20120710 PubMed PMID: 22781695; PubMed Central PMCID: PMCPMC3888062. doi: 10.1038/nbt.2287 22781695 PMC3888062

[64] Hastie E, Grdzelishvili VZ. Vesicular stomatitis virus as a flexible platform for oncolytic virotherapy against cancer. J Gen Virol. 2012;93(Pt 12):2529–2545. Epub 2012/10/12 PubMed PMID: 23052398; PubMed Central PMCID: PMCPMC4091291. doi:10.1099/vir.0.046672-0. 23052398 PMC4091291

