# Supplementary material for: Cyclin-dependent kinase 5 negatively regulates antiviral immune response by disrupting myeloid differentiation primary response protein 88 self-association
Source: Virulence. 2023 Jun 18;14(1):2223394. doi: 10.1080/21505594.2023.2223394 (PMC10281466; doi:10.1080/21505594.2023.2223394)
Supplement: Supplemental Material [file KVIR_A_2223394_SM9349.zip › 2223394 Supplemental material.docx]

**Supplemental material**

**Figure S1**


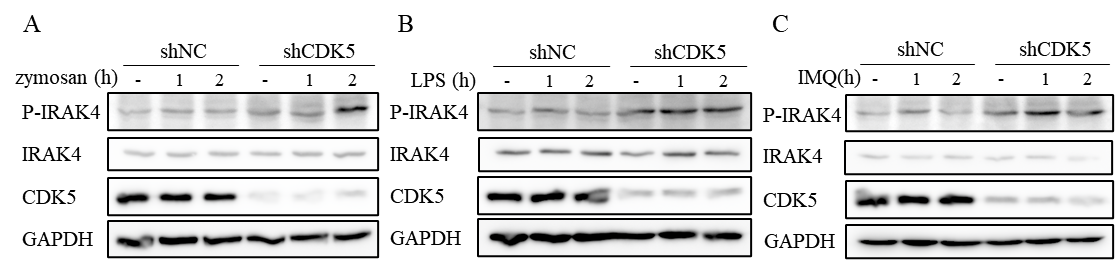


**Figure S1 Activation of IRAK4 was boosted in CDK5 deficient cells after zymosan, lipopolysaccharide or imiquimod treatment.**

shNC or shCDK5 A549 cells were treated with zymosan (100 ng/ml) (A), lipopolysaccharide (LPS) (100 ng/ml) (B), or imiquimod (IMQ) (50 ng/ml) (C) for 1 or 2 hours, then the cells were lysed for immunoblotting analysis, and the phosphorylated or total proteins were detected with specific antibodies.

**Figure S2**


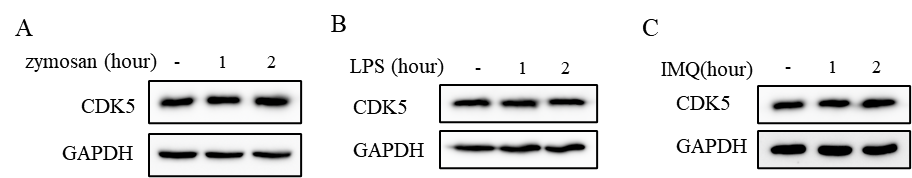


**Figure S2 The expression of CDK5 in A549 cells after zymosan, lipopolysaccharide or imiquimod treatment.**

A549 cells were treated with zymosan (100 ng/ml) (A), lipopolysaccharide (LPS) (100 ng/ml) (B), or imiquimod (IMQ) (50 ng/ml) (C) for 1 or 2 hours, then the cells were lysed for immunoblotting analysis.

**Figure S3**


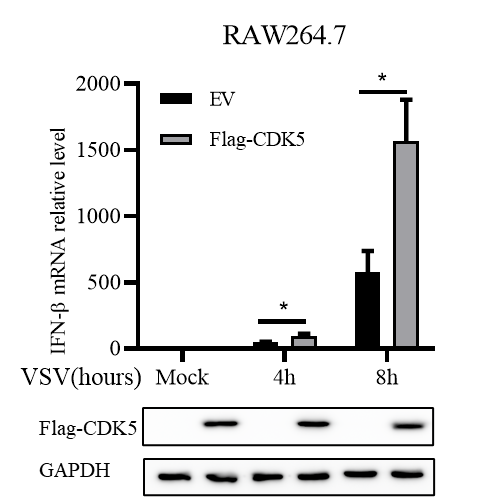


**Figure S3 The expression of IFN-β induced by VSV was increased in CDK5 overexpressing RAW264.7 cells.**

RAW264.7 cells were infected with Flag-CDK5 lentivirus for 48 hours, followed by VSV infection for 4 and 8 hours. Total RNA was extracted for qRT-PCR analysis. Graph values are presented as means ± SE, n=3. *P<0.05.
