# Supplementary material for: Cyclin-dependent kinase 5 negatively regulates antiviral immune response by disrupting myeloid differentiation primary response protein 88 self-association
Source: Virulence. 2023 Jun 18;14(1):2223394. doi: 10.1080/21505594.2023.2223394 (PMC10281466; doi:10.1080/21505594.2023.2223394)
Supplement: Supplemental Material [file KVIR_A_2223394_SM9349.zip › graph_1.docx]

**Graph 1: Proliferation assay of CCK8 experiment was performed in shNC and shCDK5 A549 cells.**

CCK-8 solution (10 µL) was pipetted into 96 well plates containing 1000 cells/well after culture of 1 day (D1),2 days (D2),3 days (D3), 4 days (D4), and 5 days (D5). Two hours after incubation, the plates were scanned with a microplate reader at 450 nm. Graph values are represented as means ± SE, n=3. *P<0.05, **P<0.01.
